# Everybody's Page

**Published:** 1889-02-16

**Authors:** 


### Everybody's Page.

The Swedish Cremation Society, founded in 1882, now numbers 3,000 members and subscribers.

---

It is said that Jerome, of Brunswick, and Hans von Gersdorf, two Alsatian surgeons, were the first writers on gunshot wounds.

---

There were 14,772 lunatics in the insane asylums of the State of New York on October 1st, 1888, as against 14,062 at the same date in the preceding year.

---

"Cardial Calisthenics" is the title of a treatment for strengthening weak heart, introduced by the Brothers Schott. It consists in a sort of wrestling match between nurse and patient, under the supervision of the physician.

---

In the early years of this century vaccination was introduced into India, and was fairly successful, though the religious scruples of the Hindus made them hesitate to accept it. Still, in the Presidency of Madras alone 178,000 persons were vaccinated in the year 1804.

---

The *Congregationalist* says that idolatry is resuming its sway in the Sandwich Islands. It assures its readers that the legislature of 1886 actually organised a society for the promotion of heathen sorcery under the innocent-sounding title of a board of health.

---

In Madame de Genlis' *Contes Moraux* there is a story about a girl who was cured of a tendency to consumption by passing six months in a cow-house, or "byre," as it is called in Scotland. This old cure is now to be tried in Berlin on a large scale. A vast circular building has been erected, in the basement of which a large number of cows is to be kept, while the odour from below is to be conducted to the rooms on the upper floors, which will be occupied by patients.

---

While the Americans are thinking of inflicting capital punishment by electricity, French medical jurists recommend a return to the old method of death by decapitation. Dr. Paul Soye, who has published a book on the subject, declares that beheading kills instantly, painlessly, and surely, and that there can be no return of consciousness after the blow falls. Yet it is recorded as noteworthy in the execution of Mary Queen of Scots, that "shee made very small noyse," and we are also told that "hir lips stirred up and downe almost a quarter of an hower after hir head was cut off." This movement does not necessarily imply consciousness, yet it is a gruesome thing to think of.

---

The *St. James's Gazette* of February 11th quoted a short paragraph from The Hospital to the effect that "children should not, if it can be avoided, wear spectacles." To this Mr. St. Clair Buxton replies in a letter to the *St. James's*, endorsing the view of The Hospital in so far as it relates to children of four years old and under, but maintaining that after that age children become so rational that they will not look over or under their spectacles, but straight through them, like grown-up persons. In our judgment children of any age, and grown-up youths and maidens too, are much better without spectacles, except in cases of absolute and proved necessity. The picture of scores of schoolgirls trooping to school and home again with spectacles on nose is not only melancholy but absurd. If the present system of education drives half the boys and girls to the use of spectacles, surely reason would say, "Change the system." To persevere with that which is distinctly and extensively injurious is neither scientific nor reasonable, but, on the contrary, both foolish and wicked.

Dr. B. Rush, a writer on insanity in the early part of the century, declares his belief that music often affords great relief in hypochondriasis. A patient of his told him that one of his paroxysms was cured by hearing "Old Hundred" sung in a country church. Luther's testimony to the power of music in quelling the devil is well known; not to mention the cure wrought in King Saul by David's music.

---

The draining of Lake Copais, which occupies a part of Attica and Beotia, in Greece, is a measure of great importance for the agriculture of that country. It is an immense shallow basin covering about 25,000 acres, and the bottom is formed of excellent alluvial soil. As there is plenty of rain in the district there is no danger of the lake being transformed, as has sometimes happened, into an arid plain.

---

A medical service seems to have been attached to the army from the remotest historical times. Barbarous tribes, indeed, neither gave nor received quarter, therefore after their battles there were no wounded needing aid. But with advancing civilization there always comes increased regard for human life, and we learn from Homer that in the camp of the Greeks before Troy great care was expended on the healing and nursing of the wounded. Xenophon also furnishes us with details concerning the medical service during the famous retreat of the ten thousand, while all extant histories go to prove that this branch of his army was an object of constant solicitude of Alexander the Great.

---

*Fiat experimentum, sat est*, said a hundred and twenty French physicians, who were anxious to believe that antipyrin prevented sea sickness. They were sailing from Marseilles to Oran, where a great medical meeting was to be held, and they set out full of hope and scientific interest. But as the wind freshened and the sea roughened, these things disappeared. They took anti-pyrin, but they took it in vain. Only fifteen of the hundred and twenty appeared at dinner, and of these only four managed to sit out the meal and profit by it. Therefore, there are now at least one hundred and sixteen French physicians who do not believe that anti-pyrin can prevent sea-sickness. And probably the other four trace their exemption to other causes than the drug. *Fiat experimentum, sat est.*

---

A writer in the American *Medical Record* has come upon an interesting archæological relic. It purports to be a "recent" book on etiquette, and gives, among other pieces of information regarding the ideas and usages of modern society, the news that the profession of medicine is not followed by gentlemen. The *Record* remarks on this that the English medical men who have visited their country have conducted themselves in such a way as to be taken for gentlemen by their American hosts. We may add that even in England the great majority of medical men have the manners, customs, and ideas of gentlemen, and would be greatly surprised to learn that they were not regarded as such. Still, we have to face the fact that they are not so regarded by the anonymous apostle of good form, whose lucubrations have surprised our American brother. But the real question at issue is, what, in the opinion of the apostle aforesaid, is a gentleman? Perhaps the definition would be that of the simple though snobbish damsel, who held that "a gentleman was a man who hadn't to work for his living." If this explanation be accepted, we must admit that medical men are at once put out of court. This is sad, but as the same definition excludes an enormous number of educated and refined men in all professions, including the present Prime Minister and most of his colleagues, the doctors are excluded in good company.

